# Pathogen diversity in mosquitoes (Diptera: Culicidae) from the Republic of Kosovo: a two-year cross-sectional study

**DOI:** 10.1016/j.nmni.2026.101806

**Published:** 2026-07-09

**Authors:** Tanto Situmorang, Betim Xhekaj, Maria Sophia Unterköfler, Lisa Schlamadinger, Karin Sekulin, Ina Hoxha, Adelheid G. Obwaller, Julia Walochnik, Edwin Kniha, Nesade Muja-Bajraktari, Kurtesh Sherifi, Hans-Peter Fuehrer

**Affiliations:** a Department of Biological Sciences and Pathobiology, University of Veterinary Medicine, Vienna, 1210, Austria; b Faculty of Agriculture and Veterinary, University of Prishtina, Bulevardi ‘Bill Clinton’, Prishtina, 10000, Republic of Kosovo; c Armaments and Defence Technology Agency, Vienna, 1190, Austria; d Center for Pathophysiology, Infectiology and Immunology, Institute of Specific Prophylaxis and Tropical Medicine, Medical University Vienna, Kinderspitalgasse 15, Vienna, 1090, Austria; e Division of Science, Research and Development, Federal Ministry of Defence, Roßauer Lände 1, Vienna, 1090, Austria; f Department of Biology, Faculty of Mathematics and Natural Sciences, University of Prishtina, Mother Theresa Street, Prishtina, 10000, Republic of Kosovo

**Keywords:** Mosquito-borne pathogens, Filarioidea, Plasmodium, Trypanosomatidae, Kosovo, Molecular surveillance

## Abstract

**Background:**

Mosquitoes (Diptera: Culicidae) are important vectors of human and animal pathogens. Balkan countries in the southeastern part of Europe exhibit a diverse mosquito fauna and can act as a crossway for both natural and anthropogenic introduction. Some countries however lack data on mosquito fauna distribution, diversity, and pathogen circulation.

**Methods:**

mosquitoes were trapped in all seven districts of Kosovo in July and August of both 2022 and 2023, then were investigated for the presence of pathogens using PCR-based methods. A total of 144 different locations were surveyed using up to three CDC miniature light traps. Individual trapped mosquitoes were morphologically identified, then pooled, and DNA/RNA was isolated. Identification was confirmed by *cytochrome oxidase I* (*COI*) barcoding, and PCR-based pathogen screening was performed, targeting the medically relevant Filarioidea, *Plasmodium* and trypanosomatid protozoans as well as arboviruses (West Nile, Dengue and Zika virus).

**Results:**

Altogether 6491 mosquitoes belonging to 18 species in six genera were trapped, identified, and screened in 1122 pools. Of 986 female pools, 21 pools (2.1%) tested positive for DNA of filarioid helminths (*Dirofilaria immitis* and *D. repens*), 52 pools (5.3%) tested positive for avian *Plasmodium* DNA, and 82 (8.3%) tested positive for trypanosomatid DNA.

**Conclusion:**

This study thus presents the most extensive survey of mosquitoes and their parasites in Kosovo to date, providing data on the prevalence of the animal and human pathogens, Filarioidea, *Plasmodium* and *Trypanosoma*.

## Highlights

• First nationwide molecular survey of mosquitoes and their pathogens in Kosovo.
• Eighteen mosquito species identified from six genera across all seven districts.
• Filarioidea, avian *Plasmodium*, and trypanosomatids detected in mosquito pools.
• COI barcoding combined with PCR enabled accurate species and pathogen detection.
• Provides baseline data on mosquito-borne pathogens in a Balkan crossway region.

## 1 Introduction

Mosquitoes (Diptera: Culicidae) are important vectors of human and animal pathogens, such as West Nile Virus, the protozoan parasites *Plasmodium*, or filarioid nematodes of the genus *Dirofilaria* [1]. In Europe, various endemic species of the genera *Anopheles*, *Aedes*, and *Culex* are of medical and veterinary importance, acting as reservoirs and vectors of zoonotic pathogens to humans [2]. In addition, the local European mosquito fauna has been re-shaped by the introduction of non-endemic species such as *Aedes* (*Stegomiya*) *albopictus* (Skuse, 1894) and *Aedes* (*Hulecoeteomiya*) *japonicus japonicus* (Theobald, 1901), which are associated with a particular risk of introduction and potential transmission of non-endemic pathogens such as Dengue virus, due to the high vector capacity of *Ae. albopictus* [3,4].

Balkan countries in the southeastern part of Europe exhibit a diverse mosquito fauna and can act as a crossway for both natural and anthropogenic introduction [5,6]. Among other Balkan countries, the Republic of Kosovo has only been extensively surveyed once, reporting thirteen species in five genera in the nation's monitoring programme in 2016 and 2017, with *Culex pipiens* s.l. being the most prevalent species [7]. In contrast to the neighboring country Albania, which reported the first introduction of *Ae. albopictus* in 1979, the presence of this species in Kosovo was not noted before 2020 [8].

To date molecular screenings for mosquito-borne pathogens are lacking for Kosovo, despite confirmed circulation in other Balkan countries such as Bosnia and Herzegovina [9] or Serbia [10]. In particular, the filarioid nematodes of the genus *Dirofilaria* pose a serious threat to both veterinary and public health [11]. They primarily parasitise dogs and cats but can also infect humans. While humans are dead-end hosts for these parasites, meaning they cannot reproduce within the human body, they can still cause health problems depending on the site of infection [12]. The transmission of filarioids relies on mosquitoes ingesting microfilariae, larvae developing to the third stage (L3) in the mosquito and being inoculated into a new host during feeding. Potential natural vectors in Europe include *Cx. pipiens* s.l., *Cx. theileri*, *Ae. vexans*, *Ae. albopictus*, *Ae. caspius*, *An. maculipennis* s.l., and *Cq. richiardii* [13,14].

Haemosporidians are obligate parasites of the phylum Apicomplexa. The genus *Plasmodium* is responsible for avian malaria worldwide. They are characterised by heteroxenous life cycles, with dipteran vectors acting as the definitive host for the sexual stages and sporogony, and vertebrate animals as the intermediate host for the asexual stages and gametocytogenesis [15,16]. These parasites infect both domestic and wild bird populations, causing clinical symptoms ranging from pale mucous membranes and dyspnoea to lethargy and pre-acute death [17]. In temperate regions, *Cx*. *pipiens* s.l. are the primary natural vectors for avian malaria [[18], [19], [20]]. One of the most common avian Plasmodium found in mosquitoes and birds in Europe is *Plasmodium relictum*, which has several different mitochondrial haplotypes, such as SGS1, GRW11 and GRW4 [21].

Trypanosomatids are parasitic flagellates that infect both invertebrates and vertebrates [22,23]. Several genera of the Trypanosomatidae family are monoxenous, with only one host, while others are dixenous, with more than one host. Dixenous species are without question more important as they are able to cause severe diseases in humans, domestic animals, and even plants [24]. Dixenous species cause diseases such as African sleeping sickness (*Trypanosoma brucei*), Chagas disease (*T. cruzi*) and leishmaniasis (*Leishmania* spp.). Conversely, monoxenous species are often considered less relevant. Only in the last decade have they been viewed as a highly diverse, adaptable, and ubiquitous group, and as a crucial evolutionary link in the emergence of a dixenous parasite life cycle [[25], [26], [27], [28], [29]]. Dissection of various bloodsucking insects’ guts found that mosquitoes carried both dixenous avian trypanosomes and monoxenous trypanosomatids, whereas biting midges were only infected with monoxenous species [30]. Mosquitoes of the genus *Culex* appear to transmit avian *T. culicavium* in Europe, whereas mammalian *T. theileri* or theileri-like parasites are mainly found in *Ae. vexans* and *Cq. richiardii* [18,26,31].

To date, no studies have investigated the presence of the pathogens mentioned above in mosquitoes in Kosovo. This study provides the first screening of mosquito-borne parasites across the country and updates the existing data on mosquito species composition.

## 2 Material & methods

### 2.1 Study area

This study was conducted in the Republic of Kosovo, a landlocked country, divided into seven districts and located in the centre of the Balkan Peninsula in South-Eastern Europe. The landscape is dominated by mountains in the west, along the border with Montenegro, in the south along the borders with Albania and North Macedonia, and in the north with Serbia. The climate is continental and is affected by the hot air masses travelling across the Adriatic Sea to western Kosovo. In the countryside, agricultural activities include the farming of various animals, such as cattle, sheep, goats, poultry and pigeons. Dogs are widely present, either as strays or as pets or shepherd dogs kept in private households.

### 2.2 Entomological sampling

**Fig. 1:**
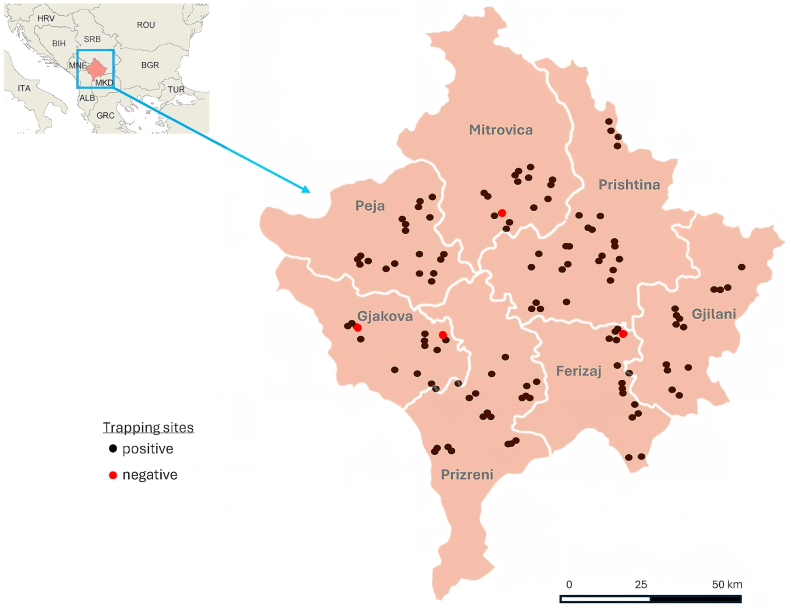
Map of Kosovo. The geographic position of Kosovo in the Balkan (top left) and seven districts of Kosovo and trapping sites. Positive sites are indicated as black dots, while negative ones as red dots.

**Fig. 2:**
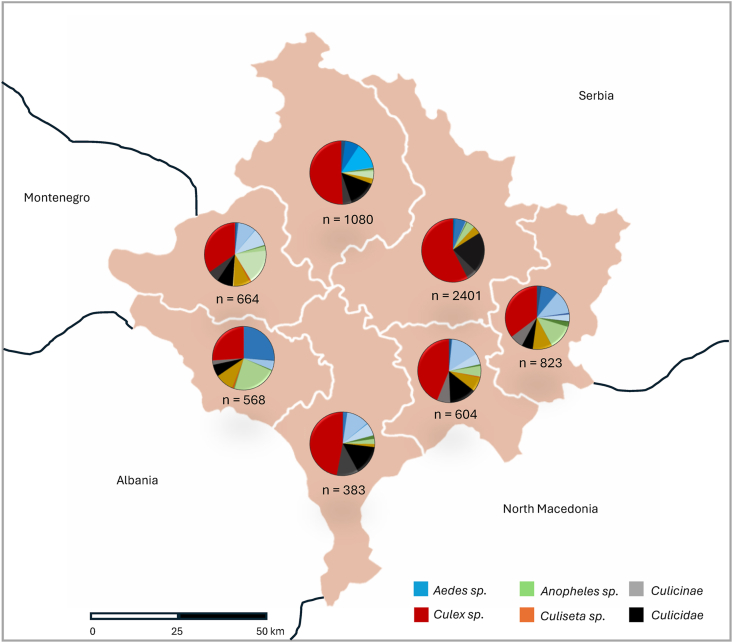
Diversity of recorded mosquito genera in seven districts of Kosovo. *Aedes* mosquitoes are represented in shades of blue, *Anopheles* in shades of green, *Culex* in red, *Culiseta* in orange, Culicinae in grey, and Culicidae in black.

An entomological survey was conducted in July and August of 2022 and 2023, in all seven districts of Kosovo. A total of 144 different locations were surveyed using up to three CDC miniature light traps (John W. Hock Company, Gainesville, FL, USA), resulting in 323 collection events. The number of collection sites was as follows: 15 in Ferizaj, 16 in Gjakova, 16 in Gjilani, 17 in Mitrovica, 30 in Peja, 35 in Pristhtina, and 15 in Prizreni district (Fig. 1). Sampling locations were chosen randomly, targeting dog shelters, cow farms, chicken farms, goat farms, sheep farms, and pigeon farms. Respective data on coordinates, farm type, and trap locations (outdoor or indoor) were always registered. Traps were deployed at sunset, operated overnight, and retrieved prior to sunrise the following morning. They were positioned 1 m above ground level. Upon collection, the nets were immediately placed on dry ice, transported to the laboratory under chilled conditions, and stored at −80°C until morphological identification (see Fig. 2).

### 2.3 Morphological identification & nucleic acid extraction

Trapped specimens were identified morphologically based on the identification keys of Becker et al., 2020 [32]. They were then sorted and pooled according to species, bloodmeal status, trapping location and date, and stored at −20°C until further analysis. The pooled specimens (1122 pools, maximum 20 specimens) were homogenized in 0.5 mL of Dulbecco's Modified Eagle Medium supplemented with 1% penicillin/streptomycin, 10 μg/mL gentamicin, 20% bovine serum albumin, and 0.25 μg/mL amphotericin B (each from Gibco, Thermo Fischer Scientific, Waltham, MA, USA). Two (3 mm diameter) metal beads were added to each 2.0 mL safe lock tube and then the sample was homogenized with a TissueLyser bead mill (QIAGEN GmbH, Hilden, Germany) for 2 min of shaking at 30 Hz. The homogenate was subsequently cleared via centrifugation for 5 min at 14,000 rpm, in a benchtop centrifuge at 4°C. To isolate whole nucleic acid, 200 μL of the supernatant was taken from the mosquito pools. Extraction was performed using the innuPREP AniPath DNA/RNA kit (iST Innuscreen, Berlin, Germany) on the KingFisher™ Apex Purification System.

### 2.4 DNA barcoding

For species-level identification, a barcoding PCR was conducted targeting a 658 base pair (bp) fragment of the subunit of the *cytochrome c oxidase subunit I* (*COI*) gene, employing the primers LCO1490/HCO2198 following Folmer et al., 2014 [33]. The sequences were uploaded in the National Center for Biotechnology Information (NCBI) sequence database (accession numbers: PX501678–PX501710) and compared with reference sequences using the Basic Local Alignment Search Tool (BLAST) in NCBI GenBank.

DNA-based pathogen detection.

All pools of samples were additionally tested by PCR for the presence of DNA of Filarioidea, *Plasmodium* spp., and *Trypanosoma* spp. (Table 1).

**Table 1:** Protocols of DNA-based pathogen detection by PCR applied in this study.

Organism Target (length)	Primer 5′-3′	Protocol	Reference
**Filarioidea**^a^*cytochrome c oxidase I* (*COI*) (∼668 bp)	COlint_F: TGATTGGTGGTTTTGGTAA COlint_R: ATAAGTACGAGTATCAATATC	94°C/2 min; 8 cycles with 0.5°C reduction/step: 94°C/45 s, 51°C/45 s, 72°C/1.5 min; 25 cycles: 94°C/45 s, 45°C/45 s, 72°C/1.5 min; 72°C 7 min	(Casiraghi et al., 2001) [34]
***Plasmodium* spp.**^b^*cytochrome b* (*cytb*) (∼617 bp)	Haem_NF1: CATATATTAAGAGAA**5**TATGGAG Haem NR3: ATAGAAAGATAAGAAATACCATTC	95°C/2 min; 35 cycles: 95°C/45 s, 50°C/45 s, 72°C/1 min; 72°C 5 min	(Hellgren et al., 2004) [15]
*cytb* (∼480 bp)	Haem_F: ATGGTGCTTTCGATATATGCATG Haem_R2: GCATTATCTGGATGTGATAATGGT	95°C/2 min; 35 cycles: 95°C/45 s, 50°C/45 s, 72°C/1 min; 72°C 5 min	
**Trypano-somatidae**^b^ 18S ribosomal RNA (∼1320 bp)	Tryp_18S_F1: GTGGACTGCCATGGCGTTGA Tryp_18S_R1: CAGCTTGGATCTCGTCCGTTGA	96°C/5 min; 35 cycles: 94°C/1 min, 56°C/1 min, 72°C/1 min; 72°C 5 min	(Peña-Espinoza et al., 2023) [35]
18S rRNA (∼960 bp)	Tryp_18S_F2: CGATGAGGCAGCGAAAAGAAATAGAG Tryp_18S_R2: GACTGTAACCTCAAAGCTTTCGCG	96°C/5 min; 35 cycles: 94°C/1 min, 56°C/1 min, 72°C/1 min; 72°C 5 min	

a Touchdown PCR.

b Nested PCR.

All PCRs were performed in a final volume of 25 μL, using either Green GoTaq Buffer (5X; Promega, USA), GoTaq Polymerase (10 U/ml) or a 2 x EmeraldAmp GT PCR Master Mix (Takara Bio Europe AB, Goteborg, Sweden). Positive samples were sent for Sanger sequencing Microsynth Austria GmbH, Vienna, Austria). Bidirectional sequences were then aligned using GeneDoc 2.7 [36]. All edited sequences were subsequently subjected to BLASTn (www.ncbi.nlm.nih.gov/blast/). Furthermore, *cytb* sequences were subjected to BLAST analysis in the MalAVI database (http://130.235.244.92/Malavi) to determine haemosporidian lineages.

### 2.5 RNA-based pathogen detection

A multiplex RT-qPCR assay specific for West Nile (WNV), Dengue (DENV), and Zika (ZIKV) Virus was conducted using designated primers and probes for each virus. The following primers were used: for WNV lineage 1 and 2 detection, WNV-8-F (5′-CGCCTGTGTGAGCTGACAAA-3′), WNV-118-R (5′-GCCCTCCTGGTTTCTTAGACATC-3′), and WNV-67-P (5′-FAM-TGCGAGCTGTTTCTTAGCACGA-BHQ1–3′) as described by Kolodziejek et al., 2014 [37]; for DENV detection, the assay incorporated DEV-F (5′-GGAAGTAGAGCAATATGGTACATGTG-3′), DENV-R (5′-CCGGCTGTGTCATCAGCATAYAT-3′), and DENV-P (5′-HEX-TGTGCAGTCCTTCTCCTTCCACTCCACT-BHQ1–3′) following Pang et al., 2014 [38]; and ZIKV-specific amplification was performed using ZIKV-F (5′-CCGCTGCCCAACACAAG-3′), ZIKV-R (5′-CCACTAACGTTCTTTTGCAGACAT-3′), and ZIKV-P (5′-Cy5-AGCCTACCTTGACAAGCAATCAGACACTCAA-BHQ2–3′) according to Fournier et al., 2023 [39], following the RT qPCR protocol published in Hoxha et al., 2025.

## 3 Results

### 3.1 Mosquito sampling, identification and barcode inventory

In total, 144 locations were sampled, of which 140 (97.2%) were positive for mosquitoes (Fig. 1). The number of sampling locations per region ranged from 15 to 35. Of 6491 trapped specimens, 5581 (86%) were female, and 911 (14%) were male. Of all females, 1065 (1065/5581; 19.1%) were engorged. Morphological identification revealed 17 mosquito species in 5 genera, namely *Aedes*, *Anopheles*, *Coquilettidia*, *Culex*, and *Culiseta*. Barcodes of all species identified were generated by *COI* sequencing and uploaded to NCBI GenBank (Table 2). The predominant species were *Culex pipiens*/*torrentium* (3052/6491; 47%), *Aedes caspius* (665/6491; 10.2%), and *Anopheles maculipennis* s.l. (535/6491; 8.2%) (Table 2). A total of 1409 (21.7%) specimens could not be identified to species level, mainly due to physical damage (e.g., loss of limbs or scales), which hindered morphological identification.

**Table 2:** Identified mosquito species, number of sequenced barcodes, accession numbers, and percent sequence identity to reference sequences in GenBank.

species	total	barcodes	accession	% identity (reference sequences)
*Ae. annulipes*/*cantans*	1	1	PX501678	100% (PV094697)
*Ae. berlandi*/*pulcritarsis*^a^	185	2	PX501686, PX501687	99.48 - 99.85% (OP709259, PP694673)
*Ae. caspius*	665	2	PX501679, PX501680	99.7 - 100% (MT708649, MH643759)
*Ae. flavescens*	2	2	PX501681, PX501682	99.67- 100% (MK403508)
*Ae. geniculatus*	11	2	PX501683, PX501684	99.85 - 100% (MW535828)
*Ae. japonicus japonicus*	1	1	PX501685	100% (GQ254793)
*Ae. vexans*	181	3	PX501688–PX501690	99.85% (MH370023, MK402895)
*An. claviger* s.l.	58	2	PX501691, PX501692	99.24 – 99.85% (PQ740514, MH643764)
*An. maculipennis* s.l.	535	1	PX501693	100% (OR200945)
*An. melanoon*	1	1	PX501694	99.7% (MH643750)
*An. plumbeus*	4	1	PX501695	100% (ON127201)
*Cq. buxtoni*	1	2	PX501696, PX501697	100% (PQ588190)
*Cq. richiardii*	16	3	PX501698–PX501700	100% (MK403239, MT731260)
*Cs. annulata*	364	4	PX501701–PX501704	100% (PQ588189, MK403505)
*Cs. longiareolata*	3	2	PX501705, PX501706	100% (MK863419, MT993479)
*Cx. modestus*	3	1	PX501707	99.85% (MK971989)
*Cx. pipiens/torrentium*^b^	3052	2	PX501708, PX501709	99.7% (OM748711, KM258189)
*Cx. theileri*	1	1	PX501710	99.69% (OL457139)

*Aedes* spp.	69		-	-
*Anopheles* spp.	31		-	-
*Culiseta* spp.	4		-	-
Culicidae	941		-	-
Culicinae	364		-	-

**Total**	**6491**			

a Barcodes match with *Ae. pulcritarsis*.

b Barcodes match with *Cx. pipiens* s.l.

### 3.2 Mosquito distribution

Species richness ranged from eight (Mitrovica) to eleven (Peja) (Table 3), of which *Ae. caspius, Ae. vexans, An. maculipennis* s.l.*, Culex pipiens/torrentium and Culiseta annulata* were found in all seven regions. Single specimens of *Ae. annulipes*/*cantans*, *Ae. japonicus japonicus*, *An. melanoon*, *Cq. buxtoni* and *Cx. theileri* were found in Prizreni, Mitrovica, Gjilani, Gjakova and Peja, respectively. The number of mosquitoes caught per district varied between 387 (Prizreni) to 2405 (Prishtina) (Table 3). Noteworthy, no Asian Tiger Mosquito (*Ae. albopictus*) was found; however, only light trapping was performed, which might not be very suitable for day-active species like *Ae. albopictus*.

**Table 3:** Number of mosquito species per district.

Species	Prishtina	Mitrovica	Peja	Prizreni	Ferizaj	Gjilani	Gjakova	Total
*Ae. anulipes*/*cantans*				1				1
*Ae. berlandi/pulcritarsis*	20	78	6	8	4	70		186
*Ae. caspius*	127	141	21	45	87	97	147	665
*Ae. flavescens*	2							2
*Ae. geniculatus*	1		1	1		6	2	11
*Ae. japonicus japonicus*		1						1
*Ae. vexans*	7	2	58	25	32	28	28	180
*An. claviger* s.l.		7	16	6	5	19	3	56
*An. maculipennis* s.l.	101	38	120	11	33	103	130	536
*An. melanoon*						1		1
*An. plumbeus*							3	3
*Cq. buxtoni*							1	1
*Cq. richiardii*	2		6		3		5	16
*Cs. annulata*	97	27	56	6	44	81	54	365
*Cs. longiareolata*			3					3
*Cx. modestus*	1				1			2
*Cx. pipiens/torrentium*	1385	550	231	182	265	292	147	3052
*Cx. theileri*			1					1

*Aedes* spp.	15	22	6		7	19		69
*Anopheles* spp.	13	10	4			4		31
*Culiseta* spp.	1	2		1				4
Culicidae	508	152	56	59	83	48	35	941
Culicinae	125	49	39	42	41	55	13	364

**Total**	2405	1079	624	387	605	823	568	6491

### 3.3 DNA-based pathogen screening

**Fig. 3:**
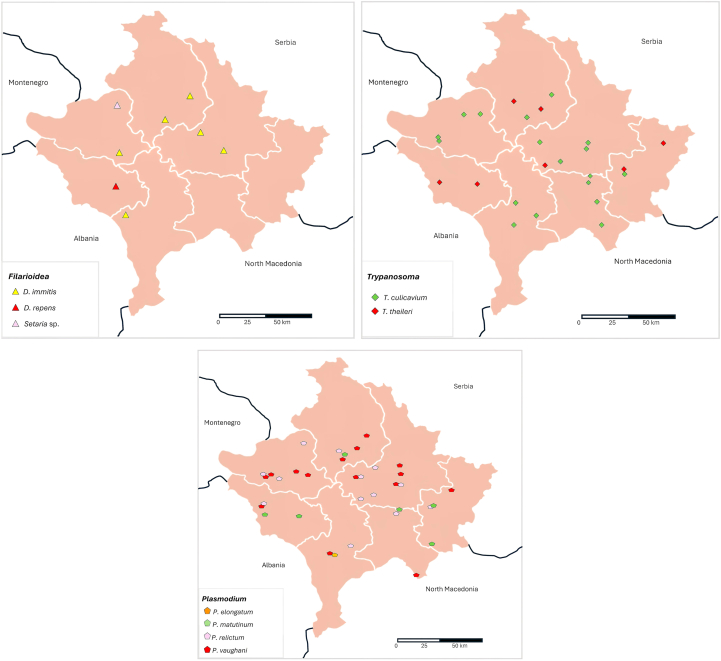
Pathogen-positive trapping sites in Kosovo. Trapping sites of mosquito pools positive for *D. immitis*, *D. repens* and *Setaria* sp. (A), *Plasmodium* spp. (B), *Trypanosoma* spp. (C).

Of 5581 female specimens tested (in 986 pools), filarioid helminth DNA was found in 21 (2.1%) pools of three different mosquito species, namely *An. maculipennis* s.l., *Cx. pipiens/torrentium* and *Ae. geniculatus*. The most prevalent Filarioidea species was *Setaria labiatopapillosa* (n = 12), followed by *Dirofilaria immitis* (n = 6), *D. repens* (n = 2), and *Setaria sp.* (n = 1). The parasite was not detected in mosquitoes from Ferizaj and Prizreni (Fig. 3). Two *An. maculipennis* s.l. pools from a location in Gjakova (Fig. 3, Table S1) were positive for *D. repens* (PX640820-21), the *COI* sequence being identical to that of a dog in Denmark (PQ772105) and of a cat in France (PQ560501). Three pools of *Cx. pipiens/torrentium* and three pools of *An. maculipennis* s.l. were positive for *D. immitis* (PX640822), with *COI* sequence identical to that isolated from a jackal in Iran (KT351849). The only *Setaria sp.* (PX640823) positive sample was found in a single *Ae. geniculatus* (Table S1).

*Plasmodium* DNA was found in 52 (5.3%) pools that solely consist of *Cx. pipiens/torrentium*. (Table S1). Six different genetic lineages of avian plasmodia were found, with the predominant being *P. vaughani* SYAT05 (n = 15, 37.5% of total infections) and *P. relictum* SGS1 (n = 14, 35%). Other lineages found were *P. matutinum* LINN1 (n = 6, 15%), *P. relictum* GRW11 (n = 3, 7.5%), *P. relictum* ARF9 (n = 1, 2.5%), and *P. elongatum* GRW06 (n = 1, 2.5%) (Table S1). *Plasmodium relictum* (PX647832-33) and *P. vaughani* (PX647834) were detected in all seven regions. *Plasmodium matutinum* (PX647835) were found in Mitrovica, Ferizaj, Gjilani, and Gjakova, while the only *P. elongatum* (PX647836) positive sample was found in a location in farm in Kema, Prizreni (Fig. 3).

Trypanosomatid DNA was found in 82 (8.3%) pools of eight different mosquito species and several pools of unidentified Culicinae and Culicidae (Table S1). Two different species of dixenous trypanosomatid parasites were identified, namely *Trypanosoma culicavium*, and *T. theileri*. Moreover, seven different species of monoxenous trypanosomatid parasites were identified, mostly *Crithidia dobrovolskii*, but also *C. brevicula*, *C. mellificae*, *Blastocrithidia culicis, Leptomonas tenua*, *Parabodo nitrophilus*, and *Rhynchomonas nasuta* (Fig. 3, Table S1). *Trypanosoma culicavium* (PX589409) were mainly detected in pools of *Cx. pipiens/torrentium* from six regions (not in Gjakova), with most 18S sequences identical to that in a *Culex* mosquito in Czechia with *Hirundo rustica* as its host (OM509727). *Trypanosoma theileri* (PX589410) were detected in one pool of *Ae. caspius*, two pools of *Cs. annulata*, and two pools of *An. maculipennis* s.l. and one pool of *An. plumbeus*. An identical 18S sequence was found in a blood sample of a cow in Texas, USA (JX178182).

No West Nile, Dengue and Zika virus RNA was detected in any female or male mosquito pools.

## 4 Discussion

This study reports the first detection of Filarioidea, *Plasmodium* spp., and trypanosomatid DNA in mosquitoes in Kosovo and substantially contributes to the further characterization of the local mosquito fauna by adding seven as yet unreported mosquito species.

To date, only one previous entomological study has been performed on mosquitoes in Kosovo, reporting 13 species, with *Cx. pipiens* s.l. being the most abundant [7]. We now present an updated and more extensive dataset with substantially more trapping sites and mosquito specimens collected. The seven additional species confirmed in our study by *COI* barcoding, were *Ae. annulipes/cantans, Ae. flavescens, Ae. geniculatus,* the potentially invasive *Ae. japonicus japonicus, An. plumbeus, Cx. modestus, and Cx. theileri*. Of note, Muja-Bajraktari et al., 2019 reported *Anopheles messeae* and *Uranotaenia unguiculata*, which were not trapped in our study. Discrepancies in trapped species can be explained by different factors. Firstly, all seven additional species reported in our study are represented by very low trapping numbers, highlighting potential underreporting in previous trappings rather than recent emergence, as all species are endemic in various regions of the Balkan peninsula [9,40,41]. Secondly, we performed light trapping, which attracts different species compared to the CO_2_ trapping performed by Muja-Bajraktari et al., 2019. This could also explain the absence of day-active species such as *Ae. albopictus* in our dataset, despite previous reports in Kosovo [8]. Thirdly, some species exhibit specialized breeding or feeding behavior, for example *An. plumbeus* breed in tree holes [40] or *Ur. unguiculata* feed on amphibians [42], thus their detection would need a more targeted trapping approach.

However, despite only a few numbers collected, some species require further discussion due to their vector competence. For example, *An. plumbeus* is a proven vector of *Plasmodium* under laboratory conditions, and while not primarily involved in malaria transmission, it has adapted to urban breeding sites and has been suspected of being responsible for locally transmitted malaria in non-endemic countries [43]. In addition, *Cx. modestus* is a known vector for West Nile (WNV) and Usutu virus [44,45], while *Cx. theileri* can be experimentally infected with WNV and transmit the virus [46]. Together with the abundant *Cx. pipiens* s.l., these species represent potential WNV vectors in the study area. Despite this, all mosquito pools tested were negative for WNV. The finding does not necessarily indicate the absence of virus circulation, as WNV prevalence in mosquito population is often low and spatially heterogeneous [47]. Indeed, WNV was recently detected in *Cx. pipiens* f. *pipiens* in a longitudinal study from a peri-urban area of Prishtina, Kosovo and also in a sporadic trapping in neighboring country Bosnia & Herzegovina [9,48], suggesting virus circulation that may not have been captured at our sampling locations or during our sampling period. Dengue (DENV) and Zika (ZIKV) were understandably not detected, since different collection methods and timing are required to trap the viruses’ capable vector *Ae. albopictus* [8].

Considering that the previous study by Muja-Bajraktari et al., 2019 had already focused on the geographical, altitudinal, and indoor vs outdoor mosquito distribution, our study aimed to present the first data on PCR-based pathogen detection and their distribution in this country. Using three separate PCRs (two nested and one touchdown) on each of the 1122 mosquito pools, the predominant pathogens detected were: *Dirofilaria immitis*, *Plasmodium relictum, P. vaughani*, and *Trypanosoma culicavium.*

*Dirofilaria immitis* was detected in mosquitoes trapped at five different locations representing four regions, but not in southeastern regions of the country (Ferizaj, Gjilani, and Prizreni). The two *D. repens* DNA positive samples were *Anopheles maculipennis* s.l. pools from a location in Gjakova. The single *Setaria sp.* positive sample was amplified from *Ae. geniculatus* caught in a private property in Peja. When compared for sequence similarity in GenBank, the closest was *S. tundra*, but with only 93.5% identity.

*Plasmodium relictum* and *P. vaughani* DNA were detected in all seven regions, exclusively in *Cx. pipiens/torrentium*. Our results are in line with Schoener et al., 2017, which reported around 5% (out of >1000 mosquito pools) were positive for Plasmodium DNA in mosquitoes from eastern Austria, with the most common lineages being *P. relictum* SGS1 and *P. vaughani* SYAT05.

In our study, *T. culicavium* was detected in nineteen sampling locations, represented in six out of seven regions of Kosovo (not in Gjakova). As reported in two other studies [26,31], this parasite is found mostly in *Culex* mosquitoes aligning with that mosquito's preference for avian hosts. A laboratory experiment showed that transmission to birds occurred only when birds ingested infected mosquitoes [26]. Notably, a recent laboratory study demonstrated that one of the monoxenous trypanosomes detected, *Crithidia mellificae*, is capable of significantly reducing the survival of several bee species following infection [49].

It should be noted that PCR-based screenings were performed with pooled samples in our study. Although screening individual mosquitoes provides the most accurate resolution of parasite diversity, particularly large sample sizes require cost and labor efficient sample pooling, and are therefore performed regularly [18,19,50]. However, vector competence cannot be assessed by this method, only assumed. To demonstrate vector competence, it is necessary to provide evidence of pathogen ingestion (detected in the gut), migration (to the salivary glands), and transmission (release of viable infective stages). Conventional PCR assays may also underestimate mixed infections, which are common in the wild, because they might preferably amplify the DNA of one parasite over another [51].

The purpose of pathogen screening is to determine pathogen presence, identify potential vector species, assess zoonotic risks, and provide evidence for vector control and surveillance programs. Monitoring the genetic diversity and geographical distribution of pathogens contributes to a better understanding of their epidemiology and transmission dynamics. It is also important to identify pathogen populations that evolve independently, as they can provide insights into local adaptation to hosts and environmental conditions. Conducting parasite screenings on a regular basis is therefore crucial for tracking changes in parasite diversity and distribution, enabling timely adaptation of control strategies.

Our findings provide important baseline data for both public and veterinary health in Kosovo. Although no arboviral RNA was detected in this survey, the presence of competent vector such as *Cx. pipiens* s.l. and *Cx. modestus*, together with the recent detection of WNV in mosquitoes elsewhere in the country [48], indicates ecological conditions required for local WNV transmission are present. Furthermore, the detection of *D. immitis* and *D. repens* DNA suggests an ongoing risk for canine dirofilariasis and occasional zoonotic infections in humans, while the widespread occurrence of avian *Plasmodium* lineages may have implications for wild and captive bird populations, including those of conservation concern. These findings support the implementation of One Health surveillance framework that links entomological monitoring with veterinary and public health surveillance. This integrated approach is particularly relevant given that most sampling sites were located on farms housing livestock and companion animals. Ultimately, such framework would facilitate early detection and effective management of emerging vector-borne disease threats.

Overall, this study provides an updated baseline of mosquito diversity in Kosovo, and for the first time reports on their associated pathogens. Continued surveillance and further research on vector competence are important for improving the understanding of transmission dynamics and to guide effective vector control measures.

## Informed consent statement

Verbal consent was obtained from all homeowners to trap at their properties.

## Funding source

The study has been funded by the Austrian defence research program FORTE of the Federal Ministry of Finance (BMF) (grant number 886318).

## CRediT authorship contribution statement

**Tanto Situmorang:** Conceptualization, Formal analysis, Methodology, Writing – original draft, Writing – review & editing. **Betim Xhekaj:** Conceptualization, Writing – review & editing. **Maria Sophia Unterköfler:** Methodology, Writing – review & editing. **Lisa Schlamadinger:** Methodology. **Karin Sekulin:** Methodology. **Ina Hoxha:** Methodology. **Adelheid G. Obwaller:** Project administration, Supervision. **Julia Walochnik:** Project administration, Supervision. **Edwin Kniha:** Conceptualization, Investigation, Methodology, Project administration, Supervision, Writing – original draft, Writing – review & editing. **Nesade Muja-Bajraktari:** Methodology, Writing – review & editing. **Kurtesh Sherifi:** Methodology, Writing – review & editing. **Hans-Peter Fuehrer:** Formal analysis, Methodology, Supervision, Writing – review & editing.

## Declaration of competing interest

The authors declare that they have no known competing financial interests or personal relationships that could have appeared to influence the work reported in this paper.

## Appendix A Supplementary data

Table S1 Detailed information on pathogen positive samples (Sheet 1), and surveyed locations in Kosovo (Sheet 2).
